# Efficacy and Safety of Apatinib Treatment for Advanced Cholangiocarcinoma After Failed Gemcitabine-Based Chemotherapy: An Open-Label Phase II Prospective Study

**DOI:** 10.3389/fonc.2021.659217

**Published:** 2021-05-03

**Authors:** Ge Zhang, Shuai Gong, Lina Pang, Lixia Hou, Wei He

**Affiliations:** Department of Oncology, The First Affiliated Hospital of Zhengzhou University, Zhengzhou, China

**Keywords:** apatinib, advanced cholangiocarcinoma, prospective study, efficacy, angiogenesis

## Abstract

**Purpose:**

As a novel small-molecule vascular endothelial growth factor receptor-2 tyrosine kinase inhibitor (VEGFR2-TKI), Methylsulfonic apatinib (apatinib) exhibits a specific antitumor effect in various solid tumors *via* inhibition of angiogenesis. The present study was performed to evaluate the clinical efficacy and safety of apatinib in the treatment of advanced cholangiocarcinoma after failed gemcitabine-based chemotherapy.

**Patients and Methods:**

This was a prospective open-label phase II trial (NCT03521219). A total of 32 patients, in whom gemcitabine-based first-line chemotherapy for advanced intrahepatic cholangiocarcinoma had failed, were consecutively enrolled in a prospective, open, exploratory, and single-center clinical trial from November 2017 to November 2018. They were treated with apatinib mesylate second-line monotherapy (orally, 500 mg per day for a cycle of 28 days) until progressive disease or unacceptable toxicity. Using Response Evaluation Criteria in Solid Tumor version 1.1 (RECIST 1.1) and the Common Terminology Criteria for Adverse Events version 4.0 (NCI-CTCAE 4.0), the efficacy and adverse were evaluated, respectively. Kaplan-Meier method was used for survival analysis.

**Results:**

Twenty-six patients were enrolled in full analysis set. At the end of follow-up, two patients were lost to follow-up, 24 of 26 patients in FAS were included in efficacy analyses. For the efficacy analysis set, the objective response rate (ORR) was 20.8% [95% confidence interval (CI): 9.24–40.47%] and the disease control rate (DCR) was 62.5% (95% CI: 112.86–387.14 days). One patient (4%) showed complete response (CR), 4 patients (17%) showed partial response (PR), 10 patients (41.7%) stable disease (SD), and 9 patients (37.5%) had progressive disease (PD). Meanwhile, apatinib therapy achieved the median progression-free survival PFS was 95 days (95% CI: 79.70–154.34 days), and the median OS was 250 days (95% CI: 112.86–387.14 days). Furthermore, univariate analysis revealed that age and tumor’s anatomic location significantly affected PFS (P < 0.05). The most common clinically adverse events (AEs) included myelosuppression (69.2%), hypertension (57.7%), proteinuria (46.2%). The AEs were mild, mainly in grade 1 or 2, and no toxicity-induced death occurred.

**Conclusion:**

Apatinib monotherapy is an effective and promising regimen for treating patients with advanced cholangiocarcinoma who experienced failure of gemcitabine-based chemotherapy.

## Introduction

Cholangiocarcinoma is a highly malignant tumor with poor prognosis. Based on the anatomic site, it is divided to intrahepatic and extrahepatic cholangiocarcinoma. The morbidity and mortality rates are high in patients aged 30 to 50 years and have been increasing recently (1). Due to insidious onset, non-specific symptoms of early cholangiocarcinoma, and lack of a particularly satisfying marker or imaging technique for diagnosis (2, 3), many cases are already in the middle-late or advanced stage at the time of the treatment, and thus, only suitable for systemic or palliative therapy. Considering that cholangiocarcinoma shows insensitivity to radiochemotherapy, surgical resection is the only means of radical therapy. However, only 10% of patients diagnosed at the early stage are eligible for surgical resection, which is why 5-year survival rate is very poor (only 5%) (4). However, although gemcitabine combined with cisplatin or 5-fluorouracil as the “gold standard” for first-line treatment has been confirmed to improve survival, the median OS time is still shorter than 1 year. Given that there is no standard second-line treatment regimen at present, it is important to urgently establish novel therapeutic methods to improve survival time and achieve low toxicity.

Recently, anti-angiogenic therapies have shown promising results. Namely, angiogenesis plays a significant role in tumor growth, metastasis, and recurrence; thus, anti-angiogenic therapy has great potential for cancer therapy (5). Considering sustained overproduction of VEGF owing to the hypoxic environment in the tumor after chemotherapy, VEGF is likely the fundamental factor promoting angiogenesis by VEGFR-mediated pathways (6). VEGFR-2, highly expressed on vascular endothelial cells and prominently mediating VEGF’s angiogenic efficacy, has become the critical target of anti-angiogenesis therapy (7, 8).

Methylsulfonic apatinib (Jiangsu Hengrui Medicine Co.,Ltd, Jiangsu, China), hereinafter referred to as apatinib, an anti-angiogenic drug with significant antineoplastic activity, was developed independently in China and approved by the China State Food and Drug Administration (CFDA) for subsequent-line therapy of advanced gastric or gastroesophageal junction adenocarcinoma (9). As a small-molecule tyrosine kinase inhibitor selectively targeting VEGFR-2, apatinib inhibits endothelial cell proliferation, reduces tumor microvascular density, and promotes cell apoptosis *via* downregulation of the VEGF pathways to achieve suppression of tumor growth and recurrence (10, 11). Apatinib has been applied for various types of malignancies and exerted obvious survival benefit with tolerable toxicity. It was specially approved as the third-line therapy in patients with advanced or metastatic chemorefractory gastric cancer (12, 13). However, there has been no definite conclusion concerning its efficacy and clinical safety for advanced cholangiocarcinoma. Here, we report an open-label phase II trial (ClinicalTrials.gov: NCT03521219) to conduct a prospective evaluation of the therapeutic effect and safety of apatinib in the second-line treatment of advanced cholangiocarcinoma.

## Patients and Methods

### Patients’ Eligibility

The inclusion criteria were as follows: 1. Male and female aged 18–75; 2. Eastern Cooperative Oncology Group Performance Status (ECOG) score of 0–2 points; 3. Histological or cytological diagnosis of recurrent or metastatic advanced cholangiocarcinoma after failure or intolerance of gemcitabine-based first-line chemotherapy regimens; 4. At least one measurable lesion by imaging examination [computed tomography (CT) or magnetic resonance imaging (MRI) ≥10 mm; CT scan thickness not greater than 5 mm], and no radiotherapy or other local therapy performed unless progression after treatment occurred (RECIST 1.1); 5. Expected life expectancy ≥12 weeks; 6. Acceptable function of vital organs: absolute neutrophil count (ANC) ≥1.5 × 10^9^/L; platelet count ≥75 × 10^9^/L; hemoglobin ≥8 g/dl; serum proteins ≥2.8 g/dl; serum total bilirubin ≤3 times upper limit of normal value (ULN), and ALT and AST ≤2.5 times ULN (if liver metastasis is present, ALT and AST ≤5 times ULN); creatinine clearance ≥50 ml/min; 7. No serious drug allergy history; 8. Subjects volunteered to participate in the study. Prior to procedures, informed consent was signed by each patient who have satisfactory compliance and can cooperate with follow-up.

The exclusion criteria were as follows: 1. Patients who had undergone targeted therapy; 2. Contraindications including active hemorrhage, ulcers, intestinal perforation, intestinal obstruction, within 30 days of major surgery, hypertension that cannot be controlled by drugs, cardiac insufficiency III or IV, and severe dysfunction of the lungs and the kidneys; 3. Coagulation disorders [international normalized ratio (INR) >1.5, thrombin time (PT) > ULN + 4 s, or activated partial thromboplastin time (APTT) >1.5 ULN), hemorrhagic tendency, or undergoing thrombolytic therapy or anticoagulant therapy; 4. Routine urinalysis suggested *urinary protein* ≥ ++ or 24-h *urinary* *protein* excretion ≥1.0 g; 5. Pregnancy and lactation; 6. Other malignancies that had been diagnosed within 5 years prior to the first use of the study drugs, except squamous cell carcinoma or basal cell carcinoma that had been effectively treated and/or carcinoma *in situ* of the cervix or breast carcinoma that had been effectively removed; 7. Other situations that may influence the conduct and outcome of the clinical research. The quitting criteria were as follows: 1. Patients who could not be treated according to the study protocol; 2. Patients who asked to quit; 3. Patients who were not fit to continue the treatment.

The approval of this study was obtained by the First Affiliated Hospital of Zhengzhou University Ethic Committee.

### Treatment

All of the patients were treated with apatinib mesylate at 500 mg orally daily, administered half an hour after the meal, for a cycle of 28 days until significant disease progression, drug intolerance, or patients’ decision. Grades 3–4 drug-related adverse events resulted in dosage reduction to 250 mg per day or interruption for several days until symptoms resolved to grade 1–2 and stabilized. Symptomatic treatments were applied with or without modification of doses for the management of toxicities during the procedure.

The comprehensive medical history and clinical and laboratory data were recorded at the beginning of the treatment. Qualified subjects entered the treatment stage after a baseline evaluation. The patients underwent CT or MRI to radiographically evaluate tumor’s response to treatment every 7 to 9 weeks during maintenance treatment, including objective tumor location and size, perineural *invasion*, lymphovascular *invasion*, and adverse events were collected. Moreover, performance status, blood pressure, blood routine parameters, urine, liver and kidney function, and electrolytes were monitored every 2 weeks.

All of the patients were followed up for 90 days to assess tumor recurrence after the last medication, undergoing a physical examination, blood testing, CT, or MRI at follow-up every 8 weeks ( ± 7 days) until disease progression, initiation of a new therapy, or death for the subjects out because of non-PD. For subjects out because of PD and those non-PD subjects completing the follow-up, a survival follow-up was carried out once per month. The enrolled patients were followed up regularly, and the drugs’ compliance and adverse events were assessed. During the follow-up, our trained clinical physicians would call them for updates (if they could not go to the hospital considering poor health following treatment).

### Efficacy and Safety Assessments

Response Evaluation Criteria in Solid Tumor version 1.1 (RECIST 1.1) criteria were used for efficacy evaluation. The patients were categorized into four groups according to tumor response: complete response (CR), partial response (PR), disease stabilization (SD), and disease progression (PD). The primary endpoint was objective remission rate (ORR), while the secondary endpoints were multiple, including the assessment progression-free survival (PFS), overall survival (OS), and disease control rate (DCR). ORR was determined as the sum of CR and PR, while DCR was calculated as the sum of the CR, PR, and SD. PFS referred to the time from the initiation of treatment with apatinib to the time of disease progression confirmed radiologically or the end of follow-up, whichever occurred first. OS was defined as the time interval between the initiation of the first treatment until death or the last follow-up date. Adverse events (AEs) were in accordance with the frequency and severity of toxicities, reported and graded by investigators on the basis of the Common Terminology Criteria for Adverse Events version 4.0 (CTCAE 4.0).

### Data Analysis

Our primary endpoint was ORR. In the setting of the initial sample size, a systematic review of Lamarca A et al. reported that the weighted mean ORR was 7.7% (95% CI: 4.6–10.9%) in the 25 studies that evaluated the use of second-line chemotherapy for advanced cholangiocarcinoma patients (including 14 phase II clinical trials, 9 retrospective analyses, and 2 case reports) (14). Under this minimum value, treatment would be considered as treatment failure. We estimated the sample size as 25 patients under an expected ORR of 20% and a minimal efficacy of 7.7% by using PASS 19. This design provided an alpha error of α = 0.05 (two-sided) and a beta error of β = 0.2 (80% power). Considering the leakage rate of 20%, we decided to enroll a total of 32 patients. All participants receiving at least one dose of apatinib were included for the analysis.

Statistical analysis was performed using the SPSS software version 19.0 (IBM, Chicago, IL, USA). Continuous variables were expressed as median (25–75th percentiles), and categorical variables were expressed as percentage (%) or number. The 95% confidence interval (CI) for the primary endpoint ORR were calculated using the Clopper-Pearson method. Kaplan-Meier method was used for survival analysis to determine median PFS and OS, with 95% CI, and the survival curve was calculated. Log- rank test was used to analyze factors affecting survival benefit. The P-value <0.05 was considered to be statistically significant.

## Results

### Patients’ Characteristics

From November 2017 to November 2018, a total of 32 patients with advanced cholangiocarcinoma were enrolled in our study and underwent apatinib therapy in the First Affiliated Hospital of Zhengzhou University. Thirty-two enrolled patients with advanced cholangiocarcinoma signed informed consent at the time we initiated this study, while six patients of them were excluded for withdrawing the consents prior to the first dose of apatinib. Ultimately, 26 patients were included in full analysis set (FAS).

Among the 26 patients included, 14 (53.8%) were men and 12 (46.1%) were women. The median age was 58 years (range: 28–78 years). ECOG 0 or 1 was present in most cases (80.8%). Twelve patients (46.2%) were diagnosed as intrahepatic cholangiocarcinoma, while 14 patients (53.8%) had extrahepatic cholangiocarcinoma. The therapy protocols were determined based on the patient’s general status, weight, age, and tolerance. Surgical resection could not be a choice in any of the 26 patients. All of the patients experienced progression after gemcitabine-based first-line chemotherapy regimens. They were prescribed with second-line monotherapy (apatinib with an initial dose of 500 mg). The most common metastasis location was liver (100.0%), followed by lungs (61.5%) and celiac lymph nodes (61.5%). Before treatment with apatinib, previous therapy was accepted by 26 patients, including combination cisplatin plus gemcitabine (n = 10), gemcitabine plus capecitabine (n = 7), gemcitabine plus fluoropyrimidine (n = 5), and gemcitabine with oxaliplatin (n = 4). The median duration of prior gemcitabine-based therapy was 4.71 ± 2.32 months (range: 1.4–8.6 months). Patient baseline characteristics at the initiation of treatment are summarized (**Table 1**).

**Table 1:** The baseline characteristics of 26 patients.

Characteristics	N	Percentage (%)
Sex		
Men	14	53.8
Women	12	46.1
Age (years)		
≤60	18	69.2
>60	8	30.8
ECOG performance status		
0	9	34.6
1	12	46.2
2	5	19.2
CA199 (U/ml)		
≤37	10	38.5
>37	16	31.5
AFP (ng/ml)		
≤400	7	26.9
>400	19	73.1
Anatomic location of the tumor		
Intrahepatic	12	46.2
Extrahepatic	14	53.8
Metastasis		
Liver	26	100.0
Lungs	16	61.5
Celiac lymph nodes	16	61.5
Pancreas	4	15.4
Adrenal gland	3	11.5
Previous therapy		
Gemcitabine plus cisplatin	10	38.5
Gemcitabine plus capecitabine	7	26.9
Gemcitabine plus fluoropyrimidine	5	19.2
Gemcitabine plus oxaliplatin	4	15.4

N, the number of patients; ECOG, Eastern Cooperative Oncology Group.

### Efficacy of Apatinib Treatment

A total of 26 patients received at least one cycle of apatinib. The follow-up continued until all the patients met PFS and OS, and the median duration of follow-up was 8.3 (range: 0.9–28.0) months. Of 26 patients, 2 were lost to follow-up, 1 did not attend the follow-up visit due to a lack of time, and 1 stopped using apatinib and dropped out for another reason. At the end of follow-up, 24 of 26 patients in FAS were included in efficacy analyses, available for assessment of efficacy, according to investigator* *assessment of targeted lesions using CT or* *MRI.

**Figure 1:**
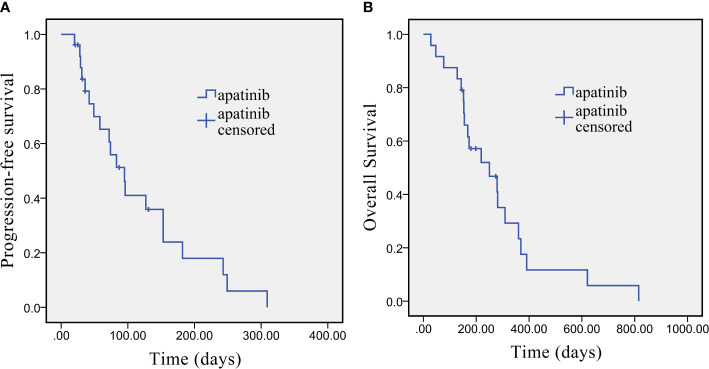
Kaplan-Meier survival curve of PFS **(A)** and OS **(B)** in advanced cholangiocarcinoma patients who underwent apatinib monotherapy as the second-line treatment.

Short-term curative effect: The evaluation of the best response was shown in accordance with RECIST v1.1. as follows: 1 patient (4.16%) showed CR, 4 patients (16.67%) showed PR, 10 patients (41.67%) had SD, and 9 patients (37.50%) had PD. The ORR was 20.8% (95% CI: 9.24–40.47%), and the DCR was 62.5% (95% CI: 42.71–78.84%). Long-term efficacy: Long-term curative effect of apatinib was analyzed by the Kaplan-Meier method for PFS and OS. The median PFS was 95 days [95% confidence interval (CI): 79.70–154.34 days], and the median OS was 250 days (95% CI: 112.86–387.14 days). Kaplan-Meier survival curves for PFS and OS are shown in **Figure 1**.

**Figure 2:**
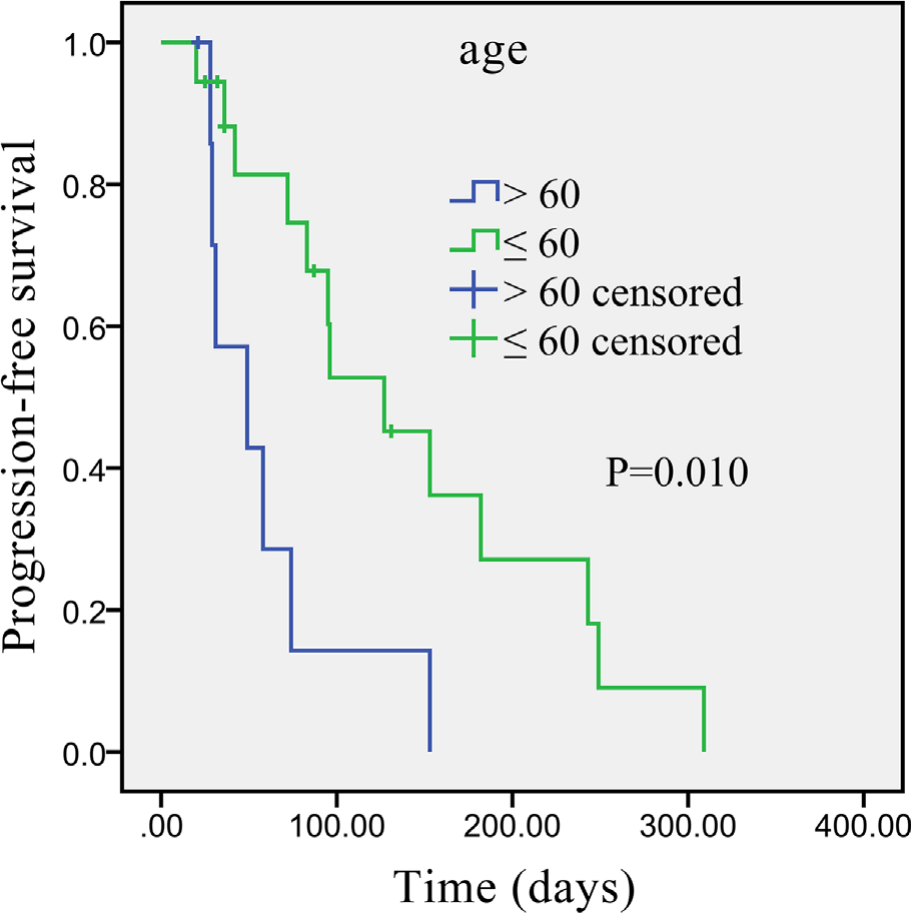
Kaplan–Meier survival curve of apatinib monotherapy as the second-line treatment, stratified by age: Survival curve shows prolonged PFS in patients ≤60 years compared with patients >60 years.

**Figure 3:**
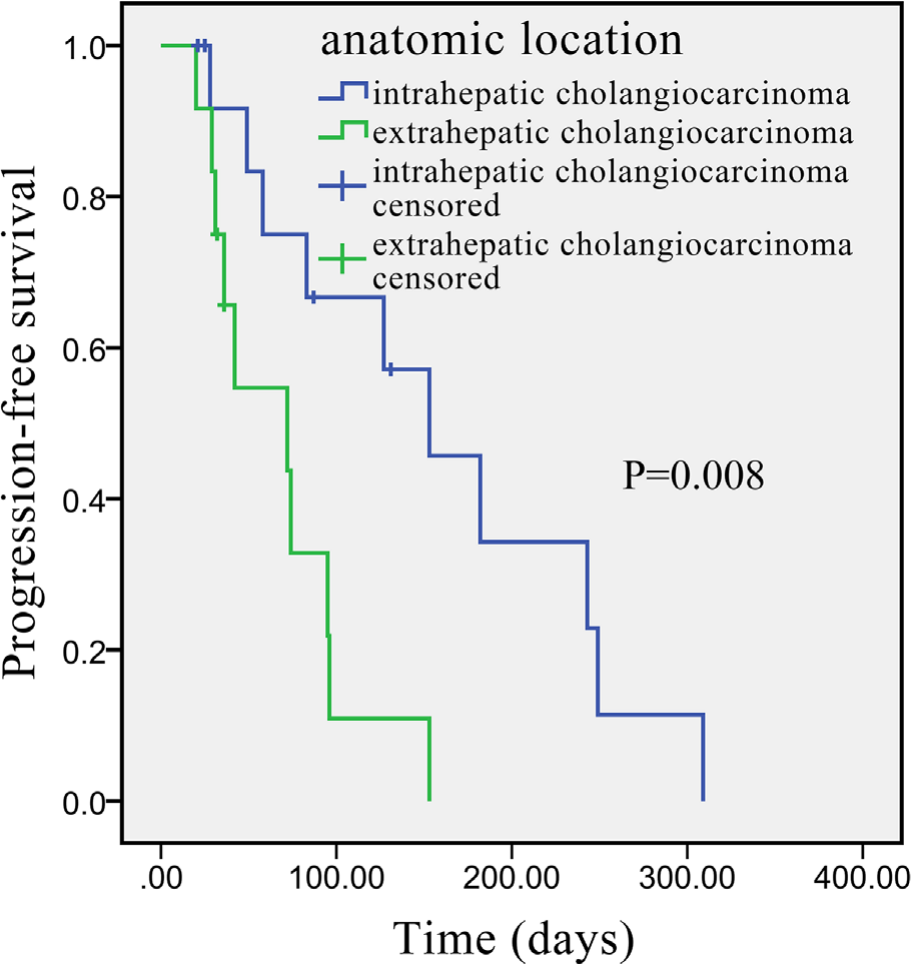
Kaplan–Meier survival curve of apatinib monotherapy as the second-line treatment, stratified by anatomic location: Survival curve indicates that the PFS was higher in patients with intrahepatic than with extrahepatic cholangiocarcinoma.

Univariate analysis revealed that some univariate factors affected the patients’ survival (**Table 2**). There was no statistically significant effect of sex. However, age was a significant independent factor correlated with PFS. The median PFS was significantly improved from 49 days (95% CI: 2.8–95.2 days) in the patients age >60 years old, to 127 days (95% CI: 64.3–189.7 days) in the patients age ≤60 years old (P = 0.010, **Figure 2**). We showed that the tumor’s anatomic location was associated with survival benefits (P = 0.08, **Figure 3**). The median PFS was 153 days (95% CI: 79.7–226.3 days) for intrahepatic cholangiocarcinoma, which was significantly longer than extrahepatic cholangiocarcinoma (72 days, 95% CI: 21.3–221.7 days). ECOG performance status and CA199 were not linked to survival benefit (P > 0.05).

**Table 2:** The log rank analysis of factors affecting PFS.

Variable	N	PFS		
		95%	CI	P
Age (years)				0.010
≤60	16	127	64.3–189.7	
>60	8	49	2.8–95.2	
Sex				0.238
Male	13	95	75.5–114.5	
Female	11	74	68.2–79.8	
CA199 (U/ml)				0.456
≤37	9	72	66.0–124.0	
>37	15	95	7.0–137.0	
ECOG performance status				0.829
0	8	127	50.0–204.0	
1–2	16	74	52.4–95.6	
Anatomic location				0.008
Intrahepatic cholangiocarcinoma	13	153	79.7–226.3	
Extrahepatic cholangiocarcinoma	11	72	21.3–122.7	

N, the number of patients; PFS, progression-free survival; CI, confidence interval.

### Safety

The safety analysis set included all 26 patients. The most frequent adverse effects are listed (**Table 3**). We observed that the majority of toxicity considered to be associated with apatinib treatment was mild, mainly classified as grade 1 or 2. One of the most common toxicities was bone marrow suppression (18/26, 69.2%), manifested as leukocytopenia and thrombocytopenia. Fifteen patients (57.7%) had secondary hypertension and 12 patients (46.2%) developed proteinuria. Hand-foot syndrome occurred in 10 patients (38.5%). Seven patients (26.9%) had fatigue, and in seven patients (26.9%) anemia was found. Liver dysfunction was observed during the treatment in some patients, including elevated transaminase (7/26, 26.9%) and elevated bilirubin (5/26, 19.2%). Rare adverse effects encompassed dizziness, nausea, and vomiting. Mild side effects were well tolerated and could be controlled by symptomatic treatment. Grade 3 adverse events included hypertension (3, 11.5%), leukopenia (3, 11.5%), thrombocytopenia (2, 7.7%), anemia (2, 3.8%), proteinuria (1, 3.8%), and elevated transaminase (1, 3.8%). None of the patients developed grade 4 toxicity. Adverse effects were manageable with symptomatic treatment or dose reduction, and there were no drug-related deaths. The initial dose of apatinib was 500 mg taken once per day and adjusted according to the patients’ intolerance. If serious adverse events occurred with intolerance in some cases, the dose was reduced to 250 mg temporarily. Dose readjustments were made after the adverse reactions had been resolved and the patients had stabilized.

**Table 3:** Main side effects of apatinib in the treatment of advanced cholangiocarcinoma.

Main side effects	I–II, N (%)	III–IV, N (%)
Secondary hypertension	12 (46.2)	3 (11.5)
Proteinuria	11 (42.3)	1 (3.8)
Thrombocytopenia	16 (61.5)	2 (7.7)
Leukocytopenia	15 (57.7)	3 (11.5)
Hand-foot syndrome	10 (38.5)	0
Anemia	5 (19.2)	2 (3.8)
Fatigue	7 (26.9)	0
ALT/AST increase	6 (23.1)	1 (3.8)
Serum bilirubin elevation	5 (19.2)	0
Anorexia	6 (23.1)	0
Oral mucositis	3 (11.5)	0
Rash	1 (3.8)	0

N, the number of patients; %, the percentage of patients.

## Discussion

Cholangiocarcinoma is a kind of highly malignant tumor originating from the biliary epithelial cells. Depending on the anatomic site, it is classified as intrahepatic and extrahepatic cholangiocarcinoma and gall bladder cancer. It is characterized by insensitivity to conventional chemotherapy and dismal prognosis with median OS of 12 months. In recent years, the morbidity rate of intrahepatic cholangiocarcinoma has shown a linear upward trend. Moreover, 60–70% of patients are at an advanced or very late stage at the time of diagnosis, and they are incurable due to few effective therapies available. Only 10% of patients are suitable to receive complete surgical resection, the only possible radical therapy, while all the remaining patients must receive palliative treatments.

Drugs approved by the FDA for non-resectable cholangiocarcinoma include gemcitabine, capecitabine, cisplatin, oxaliplatin, 5-fluorouracil, and combination therapy (15). Recently, systemic chemotherapies are considered the primary palliative treatment but with controversial efficacy. Combination Cisplatin plus gemcitabine is an appropriate option for the standard first-line chemotherapy regimen of advanced biliary cancer, based on the findings of a phase III ABC-02 study, which produced the mOS from 8.1 months prolonged to 11.7 months (16). Moreover, gemcitabine and oxaliplatin combined with erlotinib, as another therapy option, prolonged median PFS (5.9 months, 95% CI: 4.7–7.1) and hazard ratio (HR) (0.73, 95% CI: 0.53–1.00), demonstrating improved efficacy, as shown by a multicentric, randomized, phase III clinical trial (17). Meanwhile, other chemotherapy regimens like gemcitabine combined with S-1 were also recommended as first-line options (18). A part of patients in whom the first-line treatment has failed are still in good physical condition; nevertheless, no clear recommendation for the second-line treatment is available, and little research has been conducted to solve this issue. Recently, emerging outputs from the multiple phase II trials demonstrated that fluoropyrimidine-based chemotherapy, considered as the second-line treatment, benefited patients with advanced cholangiocarcinoma refractory to first-line chemotherapy (19). In recent ABC-06 trial, second-line mFOLFOX (folinic acid, 5-fluorouracil, and oxaliplatin) plus active symptom control (ASC) improved OS compared with ASC alone (12 months *versus* 6 months), providing evidence for the promising survival benefits of the use of second-line chemotherapy after progression on the cisplatin-gemcitabine combination (mFOLFOX+ASC *versus* ASC: HR 0.69, 95% CI 0.50–0.97, P = 0.031) (20).

With such modest treatment outcomes and coming of the bottleneck stage for growing study of second-line therapy strategies, a better accurate understanding of tumor biology and the underlying disease mechanisms is vital for the selecting appropriate treatment, prediction of therapy outcomes. However, a series of staging systems and therapeutic and prognostic models for cholangiocarcinoma developed so far that most incorporated independent prognostic factors such as clinical parameters and histopathological features. Hence, the reality that a need to establish more precise and robust systems and models including clinical-pathological factors, molecular and genomic information, and tumor biomarkers predicts that cholangiocarcinoma therapy now entered in era of precision medicine. The widely utilized innovative techniques and high-throughput omics technologies have led to a number of novel targeted therapy drugs and biomarkers under investigation. Critically, the selection of the appropriate biomarker, comprehension of the tumor complex molecular mechanisms will guide us as to whether targeted therapy based on genetic changes will have a future in cholangiocarcinoma. The advent of genome-wide analyses using next-generation sequencing technologies have demonstrated the landscape of molecular mutations and identified several driver genetic alterations in bile duct cancer; for example, intracholangiocarcinoma have the highest of mutations in isocitrate dehydrogenase 1 (IDH1), and fibroblast growth factor receptor (FGFR) fusions which are of special interest, because they are not detectable in other liver malignancies, whereas the most prominent mutated gene extracholangiocarcinoma is BRAF (21). Pemigatinib, as the first targeted treatment for second-line strategy approved by FDA in 2020, showed clinically relevant potential of selective FGFR1-3 inhibitor for cholangiocarcinoma (22). The inhibition of the IDH1 mutation through its inhibitor ivosidenib represents a recent breakthrough in second-line therapy for cholangiocarcinoma, dramatically improved median PFS (2.7 months *vs* 1.4 months, HR = 0.37, one-sided p < 0.0001) compared with placebo (23). As many of the targeted therapies have encouraging responses, critically, biomarker-driven clinical trials have to lay the groundwork for the best combinatorial approach of new drugs. Of note, successful approaches for targeting tumor angiogenesis have recently been worked out. Developmental angiogenesis is motivated through the interaction of VEGF and membrane receptor tyrosine kinases, including VEGFR-1, VEGFR-2, and VEGFR-3. Among those, VEGFR-2 is the most potent one involved in angiogenesis, and it supports vascular endothelial cells proliferation, migration, and survival *via* angiogenesis-mediated anti-apoptotic pathways, thus forming the basis for tumor progression and new lesions emergence (24). Consequently, many authors have suggested that reducing the overexpression of VEGF/VEGFR-2 would suppress tumor growth by prohibiting tumor angiogenesis (25). The introduction of anti-angiogenic drugs has proven to be investigated intensively as efficient subsequent options. In 2020, the first multicenter, randomized, placebo-controlled, phase II trial REACHIN reported that Regorafenib significantly increased median PFS (3.0 *versus* 1.5 months, P = 0.004) for unresectable cholangiocarcinoma in second- or subsequent-line setting, revealing that the angiogenesis drug regorafenib, as a multikinase inhibitor acting on VEGFR 1-3, platelet-derived growth factor receptor (PDGFR), FGFR, and other targets, showed good antitumor activity in biliary tumors (26). Moreover, Arkenau HT et al. used a combination of ramucirumab and pembrolizumab in 26 patients with pre-treated advanced cholangiocarcinoma, suggested that the important role of the dual inhibition of the PD-1 and VEGFR pathways, the median PFS around 1.5 months and an 11.3 months median OS (27). Although the regimens above showed underlying activity to some degree, they have been linked to a high incidence of severe adverse effects, relative lack of selectivity, as well as high cost.

A similar anti-angiogenic targeted drug, apatinib, suppressing tumor growth by highly and selectively inhibiting the tyrosine kinase activity of VEGFR-2, was approved by CFDA as early as in 2014 for advanced gastric or esophagogastric junction cancer following disease progression or recurrence after at least two previous systematic chemotherapy regimens (28). Furthermore, several phase II and III clinical trials have showcased that apatinib dramatically prolonged overall disease control rate and improved the clinical syndrome in several solid tumors, including advanced non-small cell lung cancer, breast cancer, hepatocellular carcinoma, and esophageal cancer (29–31). Overexpression of VEGF in intrahepatic cholangiocarcinoma is nearly 53%, closely associated with a worse prognosis (32). As a novel inhibitor of tyrosine kinase targeting the intracellular ATP binding site of the receptor, apatinib is capable of downregulating the activity of the RAF/MEK/ERK and PI3K/Akt signaling pathways to block the VEGF/VEGFR-2 signal conduction. In that way, it promotes apoptosis and blocks proliferation and migration of vascular endothelial cells lines, thus decreasing microvessel density of tumor and inhibiting tumor growth (33). Considering its higher binding affinity to VEGFR-2 compared with other anti-angiogenic drugs, apatinib might be able to offer further potential therapeutic opportunities for treating advanced cholangiocarcinoma, especially for patients with high expression of VEGF (34). Actually, several studies had been reported to explore the role of apatinib. A prospective open-label phase II study (NCT03251443) indicated that apatinib as non-first-line therapy has promising anti-tumor activity, with ORR 11.5%, DCR 50.0%, and median PFS 2.0 months, for the pretreated advanced biliary tract cancers (35). Likewise, in another study of second-line apatinib monotherapy, ORR was 10.0% (36).

Our study sought to prospectively analyze the application of apatinib monotherapy for advanced cholangiocarcinoma after gemcitabine-based treatment failure. We showed that the primary endpoint ORR occurred in 5 patients (20.8%) and the secondary endpoint DCR in 15 patients (62.5%). Median PFS and OS achieved 95 days (95% CI: 79.70–154.34 days) and 250 days (95% CI: 112.86–387.14 days), respectively. It is noteworthy that our treatment regimen was effective, with ORR in our study (20.8%, 95% CI: 9.24–40.47%) superior to the weighted mean ORR seen in previous studies (7.7%, 95% CI: 4.6–10.9%) according to a systematic review of Lamarca A et al. (14). The univariate analysis demonstrated significant effect of age and no significant effects of sex, ECOG performance status, and CA199 on the median PFS. Notably, we performed PFS based on the tumor site and noticed that patients with intrahepatic cholangiocarcinoma had prolonged PFS compared with patients with extrahepatic cholangiocarcinoma; thus, we speculated that apatinib monotherapy might be more beneficial for patients with intrahepatic cholangiocarcinoma. Although patients treated with apatinib achieved remarkable benefits, varied adverse effects cannot be ignored entirely during the application, including secondary hypertension, proteinuria, bone marrow suppression, hand-foot syndrome, and elevated transaminases. Most were classified as grade 1 or 2, and only a minority of patients developed grade 3 toxicities, but most of them were gradually alleviated and clinically managed after dose adjustment with optimal supportive treatment.

A number of studies indicated that the dosage in various cancer subpopulations needs adjustments to improve efficacy and reduce the incidence of adverse reactions (37). When setting the dose at 750 or 850 mg daily or 425 mg twice a day in several previous studies, most patients experienced severe adverse effects. Apatinib was well-tolerated and showed significant efficacy at doses below 750 mg per day in many solid tumors (38). Additionally, fewer AEs occurred in another study assessing apatinib for advanced cholangiocarcinoma, but similar findings have also been obtained for curative effect (rate of DCR 62.5% and the median PFS 95 days observed in our cohort *versus* the DCR rate 70.0% and the median PFS 3.0 months in that study). The difference in result might be explained by the fact that the dosage (250 mg/day) in that study was lower than in our study (500 mg/day), which may have affected the treatment response (39). Thus far, there has been no more robust evidence for the effect of the dose of apatinib set at 500 mg per day for the treatment of cholangiocarcinoma. Therefore, in the present study, we determined the initial dosage (500 mg/day) according to patients’ tolerance and general status. Despite the reduction of dosage due to intolerant toxic effects, our study’s curative effect was still maintained owing to patients’ compliance. The result suggested that to balance dose and efficacy and AEs proper dosage regimen (a daily dose of 500 mg is to start with, and subsequently decrease to 250 mg per day gradually) might be able to exert superior therapeutic benefits and diminish the rate of severe AEs. Based on these findings, we hopefully provided a reference for the use of appropriate dose and medication cycle of apatinib as the second-line therapy for advanced cholangiocarcinoma.

Although major improvement has been achieved through modern molecular profiling, most results from clinical trials using targeting angiogenesis therapies have remained limited, thus highlighting the necessity to enhance a better understanding of tumor biology, and augment therapeutic potency by combining apatinib with other therapies (40). Transarterial chemoembolization (TACE) is a commonly used intra-arterial therapy for unresectable cholangiocarcinoma, which applies minimally invasive techniques to selectively insert a catheter into the artery supplying blood to the tumor and then injects chemotherapeutic drugs and embolic agents (41). In particular, chemotherapy achieves a high dose of cytotoxic payload and block tumor-feeding arteries by killing the tumor cells based on the cytotoxic effect, while at the same time, TACE causes a microenvironment in a state of ischemia and hypoxia in the embolized tissues, which further promotes tumor angiogenesis *via* higher expression of proangiogenic factors, such as VEGF (42). However, anti-angiogenic drugs block the growth of tumor vasculature and increase the cytotoxic drug concentration, and at a certain concentration, two therapies may have a synergistic rather than just additive effect. Accordingly, anti-tumor activity is very likely to be significantly enhanced in the combination of TACE treatments and apatinib in advanced cholangiocarcinoma therapy. One retrospective study in 35 cholangiocarcinoma patients provided evidence that the combination of apatinib and TACE has efficacy in improving survival profiles. Subgroup analysis indicated that both mOS (14.0 months *versus* 6.5 months, P = 0.001, χ2 = 10.085) and mPFS (10.3 months *versus* 4.5 months, P = 0.003, χ2 = 8.835) were prolonged in the apatinib plus TACE group compared with the apatinib alone, and further univariate Cox’s regression analysis yielded that apatinib plus cTACE (*vs.* apatinib) were associated with increased mPFS (HR = 0.196, P = 0.004) and mOS (HR = 0.013, P < 0.001) (43). The introduction of immunotherapy altered the treatment regimens of various solid tumors, marking the era of modern cancer care (44, 45). A retrospective analysis suggested that the PD-1/PD-L1 pathway may be vital to the progression of unresectable intrahepatic cholangiocarcinoma in a cohort of 320 patients (45). In a combinatorial regimen randomized phase-2 study of the PD-L1 inhibitor, atezolizumab, and the small molecule MEK-inhibitor, cobimetinib in 77 patients with PD-L1 positive cholangiocarcinoma, the combination met its primary endpoint with a median PFS 3.65 months *versus* 1.87 months in the atezolizumab cohort (46). In this context, the anti-angiogenic therapy could be augmented by the immune checkpoint inhibitors by taking into account the established role of the immune system and hepatic microenvironment. Consequently, concurrent administration of apatinib with different therapy schemes as the standard practice is likely to be more therapeutically beneficial for advanced cholangiocarcinoma through the integrated control effect of systemic and locoregional therapy. In order to elucidate the synergistic efficacy and confirm the optimal mode of combination of apatinib, additional clinical studies are required in the future.

Notably, in another phase II clinical trial, for intermediate and advanced hepatocellular carcinoma patients, the patients receiving apatinib as the first-line therapy exhibited a tremendous potential in the long-term curative effect (mPFS: 8.7 months; mOS:13.8 months) (47). Therefore, we intend to conduct a new prospective study to fill the gap that there is a limited related theoretical basis for the therapeutic efficacy of apatinib in the first-line treatment of recurrent or metastatic advanced cholangiocarcinoma in the future.

There were a few limitations in our study. First, this study was a single-center trial, with the lack of a concurrent control arm. Second, the population of sample size was very small. Third, a portion of patients withdrew the consents, quit the trial before efficacy assessment, or was lost to follow-up, which might affect the real efficacy. Furthermore, we could not further analyze the clinical and molecular characteristics; this was limited due to the absence of appropriate biomarkers of anti-angiogenic agents. Therefore, large randomized controlled trials from multi-center with large sample sizes will include biomarkers investigation to predict the clinical value of apatinib in advanced cholangiocarcinoma.

## Conclusion

Taken together, anti-angiogenesis plays an essential role in anti-tumor therapy. Improved efficiency of VEGFR blocking renders apatinib one of the practical and safe concurrent treatments in advanced cholangiocarcinoma detected initially, following failure of gemcitabine-based first-line chemotherapy. Further large-scale prospective and adequately powered clinical trials should be conducted to verify apatinib’s effectiveness and safety as a second-line therapy, especially to explore more advantageous effects of combination therapies on cholangiocarcinoma and clarify the optimal therapeutic duration, dosage, clinical combination mode, and underlying mechanisms of apatinib regimen.

## Data Availability Statement

The raw data supporting the conclusions of this article will be made available by the authors, without undue reservation.

## Ethics Statement

The studies involving human participants were reviewed and approved by First Affiliated Hospital of Zhengzhou University Ethic Committee (NO.SS-2018-08). The patients/participants provided their written informed consent to participate in this study.

## Author Contributions

GZ collected the case data and drafted the manuscript; SG and WH were responsible for patient treatment and follow-up; GZ, LP and LH were involved in the acquisition of data and the search of the literature; WH made contributions to design, quality control of the study, and to critical revision of the manuscript. All authors contributed to the article and approved the submitted version. GZ and SG contributed equally in this study. Data curation: SG, LP, LH. Methodology: WH. Resources: GZ, SG. Writing – original draft: GZ. Writing – review and editing: GZ, WH.

## Funding

This work was funded by National Undergraduate Training Programs for Innovation and Entrepreneurship. Grant Number: 202010459182.

## Conflict of Interest

The authors declare that the research was conducted in the absence of any commercial or financial relationships that could be construed as a potential conflict of interest.
